# Provision of post-crash first aid by traffic police in Dar es Salaam, Tanzania: a cross-sectional survey

**DOI:** 10.1186/s12873-018-0199-9

**Published:** 2018-11-20

**Authors:** Gift G. Lukumay, Menti L. Ndile, Anne H. Outwater, Dickson A. Mkoka, Mojgan Padyab, Britt-Inger Saveman, Susann Backteman-Erlanson

**Affiliations:** 1 Department of Community Health Nursing, Muhimbili University of Heath and Allied Sciences, Dar es Salaam, Tanzania; 2 Umeå University, Umeå, Sweden

**Keywords:** Traffic police, Post-crash care, Road traffic injury

## Abstract

**Background:**

The availability of prehospital trauma care is an important means of reducing serious injuries and fatalities associated with road traffic injuries (RTIs). Lay responders such as traffic police play an important role in the provision of prehospital trauma care to RTI victims, especially where there is no established prehospital care system. Therefore, the objective of the present study was to investigate knowledge, self-reported practice, and attitudes toward post-crash first aid among traffic police officers in Tanzania.

**Method:**

A cross-sectional survey was conducted in Dar es Salaam, Tanzania between July–September 2017 to investigate knowledge, self-reported practice and attitude among traffic police officers during provision of post-crash care. We used simple random technique to recruit 340 traffic police officers, self -administered questionnaires were used to collect data. The researchers used descriptive statistics and Pearson’s chi-square tests to analyze the data.

**Results:**

A total of 340 traffic police officers were surveyed. Nearly two thirds (65.3%) reported having had post-crash first aid on-the job training; a slightly larger proportion (70.9%) reported that they had cared for RTI victims in the previous year. The survey responses showed that, generally, traffic police officers’ level of knowledge about post-crash first aid to RTI victims was low—about 3% of the surveyed officers possessed knowledge at a level considered good. Also, there was a statistically significant correlation between higher educational attainment and greater knowledgeability (*p* = 0.015). Almost all of the officers (96%) had a positive attitude toward providing post-crash first aid to RTI victims.

**Conclusions:**

Improved training of Tanzania traffic police officers, by means of an updated post-crash first aid curriculum and updated resources is recommended. Also, user-friendly post-crash first aid leaflets should be provided to traffic police for their reference.

**Electronic supplementary material:**

The online version of this article (10.1186/s12873-018-0199-9) contains supplementary material, which is available to authorized users.

## Background

Road traffic injuries (RTIs) and deaths are a major public-health concern worldwide. It is estimated that each year about 1.25 million people die and 20–50 million more are nonfatal injured in highway accidents [1]. Among the nations of Africa, Nigeria has the greatest proportion of traffic deaths, 25%, followed by the Democratic Republic of Congo, Ethiopia, Kenya, South Africa, Tanzania, and Uganda [2]. Most of these deaths occur prior to hospitalization [3, 4]. The availability of a prehospital trauma care system (PTCS) is important if the death and disability associated with RTIs are to be reduced. A systematic review and meta-analysis of trauma systems in low and middle income countries (LMICs) found a 25% reduction in mortality risk from trauma in all areas with a PTCS [5]. Evidence shows that RTI victims benefit from access to a PTCS if fast and effective care can be provided following a potentially fatal injury that occurs in a nonclinical setting [6]. Despite this evidence, no formal system of prehospital trauma care exists in Tanzania.

In Tanzania, fewer than 10% of seriously injured patients benefit from ambulance evacuation [2]. Most of RTI victims get to the hospital facilities from the scene through the efforts of untrained civilians and medically unknowledgeable lay responders such as police officers [7, 8]. Although lay responders play a significant role in helping to transfer RTI victims from an accident scene to a health facility, they are generally not trusted by the victims to whom they provide care due to their low skills in managing casualties [9]. If effective care is to be provided, there is a need to strengthen the capacity of lay responders and for them to become competent in the provision of post-crash care [10–13]. The World Health Organization (WHO) recommends that in any area where there is no established prehospital care system, nationals should strive to strengthen lay responder care as the basis of better systems of prehospital care [14, 15].

In Tanzania, traffic police officers are officially responsible for managing the accident scene, providing necessary first aid, and transporting injured people to the hospital, either independently or in collaboration with other lay responders. Because of this responsibility, first aid is part of the curriculum during police officers’ formal training. Although traffic police officers are trained in first aid, one study of the prehospital experience of trauma patients in Tanzania found that police offices caused delays in the arrival to emergency department (ED). Apart from delays, statistics show that persons injured in road accidents were significantly more likely to die when transported to the hospital in police vehicles than in private vehicles [7]. With the intention of strengthening the traffic police officers’ first aid training, we decided to investigate baseline knowledge, self-reported practice, and attitude of traffic police officers regarding post-crash first aid to RTI victims.

## Method

### Design

A cross-sectional study was conducted to investigate current knowledge, attitudes, and practice of traffic police officers in regard to the provision of post-crash first aid to persons injured in road traffic accidents, both at the accident scene and en route to a hospital. Data were collected from July to September 2017.

### Setting/area

The present study was conducted in Tanzania’s Dar es Salaam Region, which has an area of 1590 km^2^. The region consists of three districts: Ilala, Temeke, and Kinondoni. The city of Dar es Salaam is a major commercial seaport and Tanzania’s largest city, with an estimated population of more than 5.7 million [16]. Dar es Salaam Region was selected as the setting for the present study because, according to a 2015 report by the national government, it had the nation’s highest number of road traffic incidents, accounting for more than a third of all such incidents [17].

### Sample size

We used finite population correction for proportion to calculate sample size [18]. We assumed a population proportion of 0.5 (confidence level 95%) with a margin of error (precision) of 5%. We also assumed the population of traffic police officers in Dar es Salaam to be nearly 1300, given that there were 408 traffic police officers in Ilala, 425 in Kinondoni, and 391 in Temeke. Therefore, we estimated that the sample size should be 340 traffic police officers.

### Questionnaire

A structured questionnaire was developed based on WHO prehospital care guidelines [15]. The English version of the questionnaire was translated into Swahili, the national language of Tanzania. A committee translation approach was used whereby two bilingual translators and two researchers who were also bilingual discussed the meaning of each item in the English-language questionnaire and translated it into Swahili. This approach places more emphasis on constructing and writing good questions than on simply translating the words [19]. A pilot of the questionnaire was conducted with 30 traffic police officers. In every district police headquarters, 10 traffic police officers were selected by simple random sampling for pilot-testing of the tool. The participants in the pilot study were not involved in the actual study. Following pilot-testing, two items were revised: a question about demographic characteristics and an attitudinal statement. The questionnaire consisted of 23 items:

- Ten close-ended demographic questions about age, sex, years of work experience, level of educational attainment, and experience with caring for RTI victims.
- Four open-ended practice questions (e.g., one describing a scenario in which a car and a motorcycle collide).
- Five close-ended knowledge questions about airway management, proper positioning of the victim, control of external bleeding, and fracture management.
- Four attitudinal questions about assuming responsibility, who should be responsible for initiating first aid, willingness to provide first aid, and safety in providing first aid. The attitudinal questions were answered on a 4-point Likert scale (1 = strongly agree, 2 = agree, 3 = disagree, 4 = strongly disagree).

An additional PDF file shows this in more detail (see Additional file 1: Knowledge, attitude and reported practice of Trauma First Aid questionnaire).

### Sampling procedure

Participants were proportionally selected on the basis of the number of traffic police officers in their respective jurisdictions within Dar es Salaam (i.e., Ilala, Kinondoni, and Temeke). Researchers were introduced to Regional Traffic Officers with letters from the Inspector General of Police (IGP). The Regional Traffic Officers listed all traffic points where data collection took place. Each traffic police post was visited, and all available traffic police officers were counted. Folded slips of paper had been prepared that were labeled either “yes” or “no,” their number based on the number of traffic police officers. Half of the slips read “yes” and half read “no.” The officers were asked to pick a slip from a box; those who picked the “yes” slips were asked if they would voluntarily participate in the present study. To maximize representation of women as well as men, the process of selecting participants was conducted among separate groups of male and female officers.

### Data collection technique

Before the questionnaire was administered to each traffic police officer selected for the present study, the officers were informed about the purpose of the study and told that participation was voluntary. Respondents were requested to complete the questionnaire which was estimated to take 30 min and then return it to the researcher. Participants who were not able to complete the questionnaire due to emergency activity were asked to provide their telephone number; the questionnaire was administered to them when they were not busy.

### Data analysis

Data were entered into Epi-info software version 7.2, then transferred to Statistical Package for the Social Sciences (SPSS) database program version 24 for analysis. The data cleaning procedure was conducted to identify missing items in the data set. Independent variables such as age, sex, work experience, and education level were analyzed in terms of frequency and percentage. In bivariate analysis, Pearson’s chi-square test was used to assess the association between categorical independent variables (age group, education level, and work experience) and outcome variable (knowledge, self-reported practice, and attitude toward the provision of initial post-crash care to RTI victims). We categorized participants’ knowledge on post-crash first aid into three categories, so that five correct responses out of a possible five represented “good knowledge,” three to four meant “fair knowledge” and two or less indicated “low knowledge.” For every question there was only one correct answer. Regarding reported practice, responses were coded, then categorized into correct and incorrect practice. Attitudinal statements were grouped into two categories so that “strongly agree” and “agree” represented “positive attitude” and “disagree” and “strongly disagree” were considered to indicate a “negative attitude.” Statistical significance was considered to exist at *p* < .05.

## Results

### Demographic characteristics of the participants

All 340 of the selected traffic police officers agreed to participate. Nearly three quarters of the participants were male. One third of the participants (33.8%) were in the 30–39 years age interval. The mean age was 38.7 years (*SD* = 9.3). About two thirds of the participants had completed their education to the ordinary secondary school level. Regarding work experience, 83.3% had worked between 5 and 29 years; there was a fairly even distribution among the 5-year increments within that larger grouping. About two thirds of the officers had received on-the-job first aid training. Among officers who reported providing care to RTI victims, almost 40% said they had cared for more than six RTI victims in the previous year (see Table 1).

**Table 1:** Demographic characteristics of the study participants (*N* = 340)

Characteristics	*n*	%
Sex		
Male	248	72.9
Female	92	27.1
Age group (years)		
20–29	66	19.4
30–39	115	33.8
40–49	109	32.1
50–59	50	14.7
Highest educational attainment		
Primary	34	10.0
Ordinary secondary	239	70.3
Advance secondary	23	6.8
College	33	9.7
University	11	3.2
Work experience (years)		
1–4	47	13.8
5–9	72	21.2
10–14	57	16.8
15–19	44	12.9
20–24	49	14.4
25–29	61	17.9
≥ 30	10	2.9
Previous on-the job first aid training		
Yes	222	65.3
No	118	34.7
Number of RTI victims cared for, previous 12 months		
0	99	29.1
1–2	53	15.6
3–5	54	15.9
≥ 6	134	39.4

*Note RTI =* road traffic injury

#### Knowledge

**Fig. 1:**
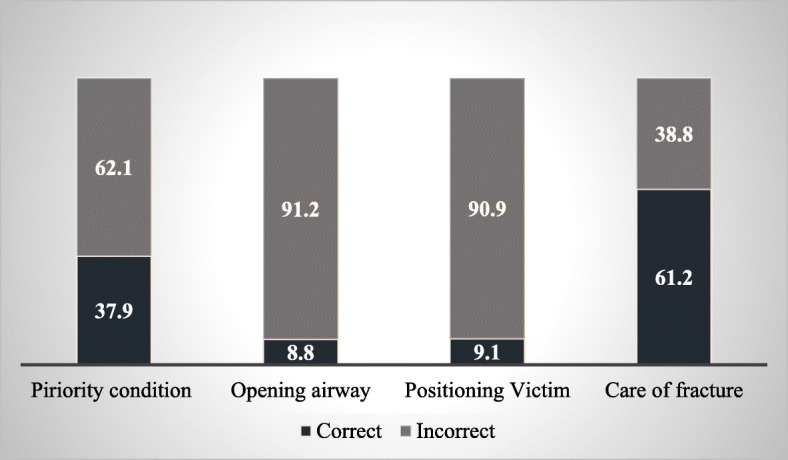
Knowledge of traffic police about managing road traffic injury (RTI) victim at the scene. Responses of traffic police officers on asked priority condition when caring for RTI victims and recommended ways of managing those conditions

About two thirds of the traffic police officers (*n* = 224; 65.9%) gave the correct answer to a question pertaining to bleeding management. Concerning fracture management, a large majority of the officers (*n* = 208; 61.2%) gave the correct answer. However, the respondents did much worse in regard to positioning a victim after a road crash: Less than 10% (*n* = 9.1) gave a correct answer. They also did poorly when asked about correct airway management: Only 8.8% (*n* = 30) provided the correct answer. When identifying priority conditions for the care of mass causalities, slightly more than one third (*n =* 129; 37.9%) of the officers gave the correct response (see Fig. 1).

**Fig. 2:**
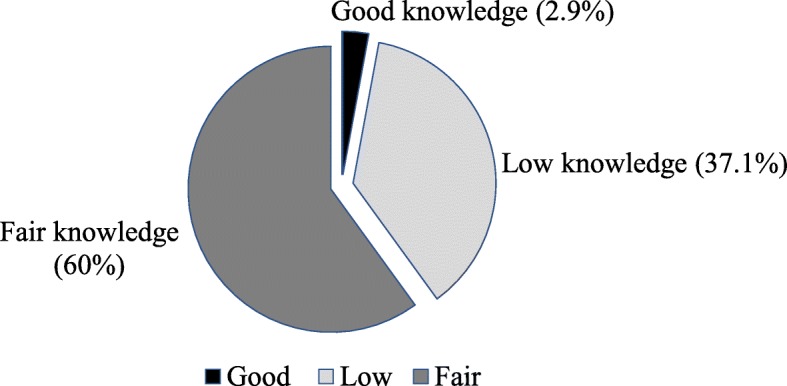
Knowledge about post-crash first aid for road traffic injury (RTI) victims. Knowledge categories of traffic police officers during provision of post-crash care to RTI victims

About one third of the participants (*n* = 126; 37%) had a low level of knowledge about caring for RTI victims, though no participants scored 0 (see Fig. 2).

### Associations between demographic characteristics and knowledge about caring for RTI victims

We compared age group, education level, and work experience with knowledge categories about caring for RTI victims to find if there were any associations. No associations were found for the demographic characteristics except for educational attainment: Participants with higher education levels had better knowledge about post-crash first aid than the others (see Table 2).

**Table 2:** Traffic police officers’ levels of knowledge about post-crash first aid (*N* = 340)

Officer characteristics	Level of knowledge		
Age group, years (*p* = .709)	Good	Fair	Low
20–29 (*n* = 66)	2 (3.0%)	42 (63.6%)	22 (33.3%)
30–39 (*n* = 115)	5 (4.3%)	72 (62.6%)	38 (33.0%)
40–49 (*n* = 109)	2 (1.8%)	63 (57.8%)	44 (40.4%)
50–59 (*n* = 50)	1 (2.0%)	27 (54.0%)	22 (44.0%)
Highest educational attainment (*p* = .015)			
Primary school (*n* = 21)	0 (0%)	1(4.8%)	20(95.2%)
Ordinary secondary school (*n* = 239)	6 (2.5%)	144 (60.3%)	89 (37.2%)
Advanced secondary school (*n* = 23)	0 (0%)	17 (73.9%)	6 (26.1%)
College (*n* = 33)	3 (9.1%)	20 (60.6%)	10 (30.3%)
University (*n* = 11)	1 (9.1%)	9 (81.8%)	1 (9.1%)
Work experience, years (*p* = .354)			
< 5 (*n* = 47)	1 (2.1%)	32 (68.1%)	14 (29.8%)
5–9 (*n* = 72)	3 (4.2%)	43 (59.7%)	26 (36.1%)
10–14 (*n* = 57)	3 (5.3%)	37 (64.9%)	17 (29.8%)
15–19 (*n* = 44)	0 (0%)	30 (68.2%)	14 (31.8%)
20–24 (*n* = 49)	0 (0%)	24 (49.0%)	25 (51.0%)
25–29 (*n* = 61)	3 (4.9%)	33 (54.1%)	25 (41.0%)
≥ 30 (*n* = 10)	0 (0%)	5 (50.0%)	5 (50.0%)
Previous on-the-job first aid training (*p* = .214)			
Yes (*n* = 222)	4 (1.8%)	133 (59.9%)	85 (38.3%)
No (*n* = 118)	6 (5.1%)	71 (60.2%)	41 (34.7%)

#### Reported practice

Concerning self and victim safety, more than two thirds of the traffic police officers (*n* = 241; 70.9%) reported engaging in the recommended practice: wearing gloves during the provision of care to the RTI victim. Regarding restoration of a compromised airway, only 4.7% (*n* = 16) officers reported applying the correct practice: the chin lift/head tilt maneuver. Concerning a question about controlling bleeding from an injured site: 81.8% (*n* = 278) reported using the correct procedure: applying direct pressure to the bleeding area with a cloth or bandage. In regard to initial management of a fractured bone, nearly half of the officers (*n* = 158; 46.8%) reported using the correct practice: applying a splint (see Table 3).

**Table 3:** Post-crash first aid practices among traffic police officers reported singly or multiply in a given scenario

Question	Responses	Officers’ responses n (%)
How would you ensure personal safety during RTI victim care	Put on gloves^a^	241(70.9)
	Put on plastic bags	37(10.9)
	Cordon off the scene	15(4.4)
	I don’t know	63(18.5)
How would you initially manage a compromised airway	Mouth to mouth breath	45(13.2)
	Chin lift head tilt^a^	16(4.7)
	Loosen belt	51(15)
	Decongest the area	92(27.1)
	Uncover the victim’s chest	64(18.8)
	Pour water on the victim	5(1.5)
	Fan the victim	74(21.8)
	Compress the chest	46(13.5)
	Rush victim to hospital	29(8.5)
	Place victim on back	66(19.4)
	I don’t know	37(10.9)
How would you control bleeding from an injured site	Applying pressure with cloth/bandage^a^	278(81.8)
	Pour cold water to injured site	4(1.2)
	Cover bleeding area with cloth/bandage	22(6.5)
	Rush victim to hospital	20(5.9)
	I don’t know	17(5.0)
How would you initially manage a fractured bone	Applying splint^a^	158(46.5)
	Stretch bone	41(12.1)
	Tie bone with bandage/cloth	76(22.4)
	Rush victim to hospital	107(31.5)
	I don’t know	28(8.2)

^a^ Recommended practice

### Associations between recommended post-crash first aid practice and demographic characteristics

No associations were found between the traffic police officers’ reported practice and selected demographic characteristics except for age group and method of bleeding control. Officers in the 20–29 age group were significantly less likely than the older officers to give the correct response (see Table 4).

**Table 4:** Associations between recommended post-crash first aid practice and demographic characteristics

Characteristic	Use of gloves		*P* -value	Chin lift/head tilt		*P-* value	Apply pressure		*P* -value	Apply splint		*P* -value
	Correct *n* (%)	Incorrect *n* (%)		Correct *n* (%)	Incorrect *n* (%)		Correct *n* (%)	Incorrect *n* (%)		Correct *n* (%)	Incorrect *n* (%)	
Age in years												
20–29	47(71.2)	19(28.8)		5 (7.6)	61(92.4)		42(63.6)	24(36.4)		27(40.9)	39(59.1)	
30–39	82(71.3)	33(28.7)	.971	3 (2.6)	112(97.4)	.283	99(86.1)	16(13.9)	.000*	58(50.4)	57(49.6)	.561
40–49	78(71.6)	31(28.4)		4 (3.7)	105(96.3)		95(87.2)	14(12.8)		48(44.0)	61(56.0)	
50–59	34(68.0)	16(32.0)		4 (8.0)	46 (92.0)		42(84.0)	8 (16.0)		25(50.0)	25(50.0)	
Educational attainment												
Primary	23(67.6)	11(32.4)		0 (0)	34 (100)		25(73.5)	9 (26.5)		14(41.2)	20(58.8)	
O-level	166(69.5)	73(30.5)		13(5.4)	226(94.6)		192(80.3)	47(19.7)		110(46.0)	129(54.0)	
A-level	19 (82.6)	4 (17.4)	.621	1 (4.3)	22 (95.7)	.618	22 (95.7)	1 (4.3)	.111	10 (43.5)	13 (56.5)	.197
College	24 (72.7)	9 (27.3)		2 (6.1)	31 (93.9)		28 (84.8)	5 (15.2)		15 (45.5)	18 (54.5)	
University	9 (81.8)	2 (18.2)		0 (0)	11 (100)		11 (100)	0 (0)		9 (81.8)	2 (18.2)	
Work experience												
< 5	34 (72.3)	13(27.7)		5(10.6)	42 (89.4)		33 (70.2)	14(29.8)		24 (51.1)	23 (48.9)	
5–9	55 (76.4)	17(23.6)		1 (1.4)	71 (98.6)		58 (80.6)	14(19.4)		33 (45.8)	39 (54.2)	
10–14	41 (71.9)	16(28.1)		1 (1.8)	56 (98.2)		46 (80.7)	11(19.3)		25 (43.9)	32 (56.1)	
15–19	31 (70.5)	13(29.5)	.344	2 (4.5)	42 (95.5)	.188	40 (90.9)	4 (9.1)	.143	22 (50.0)	22 (50.0)	.687
20–24	36 (73.5)	13(26.5)		2 (4.1)	47 (95.9)		40 (81.6)	9 (18.4)		19 (38.8)	30 (61.2)	
25–29	40 (65.6)	21(34.4)		5 (8.2)	56 (91.8)		54 (88.5)	7 (11.5)		32 (52.5)	29 (47.5)	
≥ 30	4 (40.0)	6 (60.0)		0 (0)	10 (100)		7 (70.0)	3 (30.0)		3 (3.00)	7 (70.0)	

*O-level* = ordinary secondary level. *A-level* = advanced secondary level

#### Attitude toward provision of post-crash care

Nearly unanimously, the traffic police officers responded affirmatively to the statement “I believe it’s my responsibility to provide post-crash first aid”, 99.1% either strongly agreed or agreed (see Table 5).

**Table 5:** Participants attitude towards provision of post-crash first aid to RTI victims

Attitudinal statement	Officers responses n (%)
I believe it’s my responsibility to provide post-crash first aid	
Strongly agree	185 (54.4)
Agree	152 (44.7)
Disagree	1 (0.3)
Strongly disagree	2 (0.6)
I believe post-crash first aid should be initiated by lay responders	
Strongly agree	180 (52.9)
Agree	146 (42.9)
Disagree	8 (2.4)
Strongly disagree	6 (1.8)
I’m willing to provide post-crash first aid	
Strongly agree	175 (51.5)
Agree	152 (44.7)
Disagree	9 (2.6)
Strongly disagree	4 (1.2)
I believe providing post-crash first aid will increase survival chance	
Strongly agree	196 (57.6)
Agree	135 (39.7)
Disagree	4 (1.2)
Strongly disagree	5 (1.5)

## Discussion

The aim of the present study was to investigate baseline knowledge, self-reported practice, and attitude of traffic police officers regarding post-crash first aid to RTI victims. We surveyed 340 traffic police officers in the Dar es Salaam Region of Tanzania. The officers ranged in age from 20 to 59 years, nearly two third of them were between 30 and 49. Additionally, most of the officers had been educated through the ordinary secondary school level. Our finding that a large majority of the officers had had on-the-job first aid training is comparable to the findings of studies conducted in Thailand and the United States that found that more than half of police officers had prior first aid training [20, 21].

We also found that about 71% of the traffic police who participated in our survey had provided post-crash first aid to RTI victims at least once in the previous year. This finding resembles that of a study conducted in the United States showing that, 80% of law enforcement agencies in that country provided out-of-hospital emergency care routinely [22]. In other words, at least in Tanzania and the United States, it is common practice for police to have on-the-job first aid training and to be involved in providing prehospital emergency care.

Another of our findings was that although a large majority of the traffic police officers we surveyed provided post-crash first aid, their knowledge and reported practice were poor. This finding is possibly consistent with evidence that traffic accident victims in Tanzania who are transported to the ED are significantly more likely to die when transported in police vehicles than those transported by other vehicles [7]. A comparison may be made to a study conducted in the United States showing that trauma victims transported to the ED in law enforcement vehicles were no more likely to die than those transported in emergency medical service vehicles [23, 24]. In the present study, even though more than half of the surveyed traffic police officers had previous first aid training, only one out of every 57 officers displayed a high level of knowledge about post-crash first aid. By contrast, a study conducted in Thailand showed that one out of four members of the Royal Thai Traffic Police had excellent knowledge [21]. This discrepancy might be because Tanzania’s police training is based more on theory than on practice. Yet such a training approach is not sufficient to equip police officers with the knowledge and skills they need to initiate and arrange care for RTI victims. In addition, our data showed that most traffic police officers in Tanzania have attained no higher than an ordinary secondary-level education, a factor that may contribute to the knowledge gap.

Because limited data exclusively relating to traffic police post-crash first aid care are available, comparative analysis is difficult. The study done in Thailand on traffic police responses to road traffic injuries found that 45.5% of respondents knew how to maintain the victim’s airway and proper positioning [21]. In our study, we found that only 9.0% of the surveyed Tanzanian officers had such knowledge. Both the Thailand and Tanzania studies found that bleeding control knowledge among traffic police was good. However, nearly 100% of traffic police officers in Thailand knew how to control bleeding, while in the present study we found that only 65.9% of their counterparts in Tanzania had this knowledge. One area where the officers in Tanzania outshone their counterparts in Thailand was initial management of a fractured bone, two thirds (61.2%) of the surveyed officers in Tanzania knew that application of a splint was the correct response, while only one third (32.5%) of the Royal Thai officers possessed this knowledge.

Overall knowledge scores in the Thailand study were better than those found in our study. The differences between the two studies might be due to the relative availability of training resources, considering the difference in economic status between Thailand and Tanzania. The resources that can be devoted to training and the qualification levels that can be realistically required for a person to become a traffic police officer might therefore differ.

With the exceptions of ensuring personal safety and controlled bleeding, the reported practice of traffic police officers in the Dar es Salaam Region in regard to post-crash first aid care was incorrect. The poor practice reported in the present study perhaps could be attributed to poor knowledge of post-crash first aid. But while it is believed that knowledge makes good practice more likely, we nonetheless found that, for instance, 61.2% of respondents knew that splinting was what should be done for a fractured bone but only 46.5% reported knowing how to do it correctly. This finding signifies the need for a more robust way of administering first aid training to traffic police officers.

Regarding their attitude toward providing post-crash first aid, near-unanimous majorities of the traffic police officers responded positively to all statements; in particular, almost all of them (99.1%) said they felt that it was their responsibility to provide such aid. This positive attitude might have been because first aid provision is part of the traffic police officers’ job description. A study in the United States likewise found positive attitudes toward post-crash first aid among law enforcement officers [22]. However, the positive attitudes measured with these statements do not have to say anything about how to act in a given situation. This can be interpreted to imply that the traffic police officers would like to be skilled at post-crash care.

## Conclusion

The present study found low levels of knowledge and practice of post-crash first aid among traffic police officers in Tanzania’s Dar es Salaam Region, despite the provision of on-the-job training. In light of this finding, we recommend improved competency-based training with an updated post-crash first aid curriculum and resources such as manikins so that officers can improve their practical skills. Furthermore, user-friendly post-crash first aid leaflets should be provided to traffic police for reference. In additional qualitative studies should be carried out to explore their positive attitude in relation to their first aid practice at the scene.

### Limitations of the study

It is important to recognize that the present study focused on a cross-section of traffic police officers in only one region of Tanzania. Therefore, it might be difficult to generalize the findings to all traffic police officers in Tanzania. In addition, the assessed practice of the traffic police officers was self-reported and may be different from their actual practice if observed. To limit this error, the participants were informed of the importance of providing the most accurate information they could. Despite its limitations, the study has useful implications for future training and research, considering that few studies exist that exclusively assess traffic police officers’ knowledge, practice, and attitudes in regard to post-crash first aid.

## Additional file

Additional file 1: Knowledge, attitude and reported practice of Trauma First Aid questionnaire. (PDF 183 kb)

## Abbreviations

ED: Emergency department
HIC: High-income country
IGP: Inspector General of Police
LMICs: Low- and middle-income countries
MUHAS: Muhimbili University of Health and Allied Sciences
PTCS: Prehospital trauma care system
RTI: Road traffic injury
WHO: World Health Organization

## References

[1] World Health Organization. Global status report on road safety 2015: World Health Organization; 2015.

[2] World Health Organization (2013). Road safety in the WHO African region: the facts 2013.

[3] Evans Julie A., van Wessem Karlijn J. P., McDougall Debra, Lee Kevin A., Lyons Timothy, Balogh Zsolt J. (2009). Epidemiology of Traumatic Deaths: Comprehensive Population-Based Assessment. World Journal of Surgery.

[4] Murad MK, Larsen S, Husum H (2012). Prehospital trauma care reduces mortality. Ten- year results from a time-cohort and trauma audit study in Iraq. Scand J Trauma Resusc Emerg Med.

[5] Henry JA, Reingold AL (2012). Prehospital trauma systems reduce mortality in developing countries: a systematic review and meta-analysis. J Trauma Acute Care Surg.

[6] Rogers FB, Rittenhouse KJ, Gross BW (2015). The golden hour in trauma: dogma or medical folklore?. Injury.

[7] Boniface R, Museru L, Kiloloma O, Munthali V (2016). Factors associated with road traffic injuries in Tanzania. Pan Afr Med J.

[8] Chalya PL, Mabula JB, Dass RM, Mbelenge N, Ngayomela IH, Chandika AB (2012). Injury characteristics and outcome of road traffic crash victims at Bugando medical Centre in Northwestern Tanzania. J Trauma Manage Outcomes.

[9] Kuzma K, Lim AG, Kepha B, Nalitolela NE, Reynolds TA (2015). The Tanzanian trauma patients ’ prehospital experience : a qualitative interview-based study. BMJ Open.

[10] Jayaraman S, Mabweijano JR, Lipnick MS, Caldwell N, Miyamoto J, Wangoda R (2009). Current patterns of prehospital trauma care in Kampala, Uganda and the feasibility of a lay-first-responder training program. World J Surg.

[11] Murad MK, Husum H (2010). Trained lay first responders reduce trauma mortality: a controlled study of rural trauma in Iraq. Prehospal Disaster Med.

[12] Tiska MA, Adu-Ampofo M, Boakye G, Tuuli L (2004). Mock CN. A model of prehospital trauma training for lay persons devised in Africa. Emerg Med J.

[13] Jayaraman S, Mabweijano JR, Lipnick MS, Cadwell N, Miyamoto J, Wangoda R (2009). First things first: effectiveness and scalability of a basis prehospital trauma care program for lay first-responders in Kampala, Uganda. PLoS One.

[14] World Health Organization. Post-crash response: supporting those affected by road traffic crashes. Geneva: World Health Organization; 2016.

[15] Varghese M, Sasser S, Kellermann A, Lormand JD, World Health Organization (2005). Prehospital trauma care systems.

[16] Tanzania National Bureau of statistics. Tanzania total population by districts and regions 2016-2017. Tanzania National Bureau of statistics; 2017.

[17] Tanzania Police Force; Ministry of Home Affairs, National Bureau of Statistics. Ministry finance and planning Dar Es Salaam. Crime and traffic incidents statistics report: January 2015–December. 2016:2015.

[18] Singh AS, Masuku MB (2014). Sampling techniques & determination of sample size in applied statistics research: an overview. Int J Econ Commerce Manage.

[19] McGorry SY (2000). Measurement in a cross-cultural environment: survey translation issues, Qualitative Market Research. Int J.

[20] Sztajnkrycer MD, Callaway DW, Baez AA (2007). Police officer response to the injured officer: a survey-based analysis of medical care decisions. Prehospital Disaster Med.

[21] Shrestha B, Pacheun O, Boonshuyar C, Shrestha M. Response to road traffic injuries: a survey of Royal Thai Traffic Police in a Northeastern Province of Thailand. J Public Health Dev. 15(1):101–12.

[22] Hawkins SC, Shapiro AH, Sever AE, Delbridge TR, Mosesso VN (2007). The role of law enforcement agencies in out-of-hospital emergency care. Resuscitation.

[23] Band RA, Salhi RA, Holena DN, Powell E, Branas CC, Carr BG (2014). Severity-adjusted mortality in trauma patients transported by police. Ann Emerg Med.

[24] Wandling MW, Nathens AB, Shapiro MB, Haut ER (2016). Police transport versus ground EMS: a trauma system-level evaluation of prehospital care policies and their effect on clinical outcomes. J Trauma Acute Care Surg.

