# Supplementary material for: Provision of post-crash first aid by traffic police in Dar es Salaam, Tanzania: a cross-sectional survey
Source: BMC Emerg Med. 2018 Nov 20;18:45. doi: 10.1186/s12873-018-0199-9 (PMC6247529; doi:10.1186/s12873-018-0199-9)
Supplement: Supplementary file 1 — Knowledge, attitude and reported practice of Trauma First Aid questionnaire. (PDF 183 kb) [file 12873_2018_199_MOESM1_ESM.pdf]

MUHIMBILI UNIVERSITY OF HEALTH AND ALLIED SCIENCES

DEPERTMENT OF COMMUNITY NURSING

Survey on Traffic police officers' knowledge, attitude and reported practice of trauma first aid
Background information:

Researches (Gift Lukumay and Menti Ndile) would like to invite you to participate in a survey of Traffic police officers' knowledge, attitude and reported practice of trauma first aid. Please spare 20 minutes with us to answer questions in the questionnaire. In this survey your personal information will be kept confidential. Information collected will only be used for analysis and for training purpose. Thank you!

PART A: Demographic data

1. Place of working
   1. Ilala ☐ 2. Kinondoni ☐ 3. Temeke ☐
2. Working station……………….
3. Age in years.....................
4. Sex
   1. Male ☐ 2. Female ☐
5. Years of Working as traffic police officer …………….
6. Education level
   1. Primary school education ☐
   2. Ordinary secondary school education ☐
   3. Advanced school secondary education ☐
   4. College (certificate, diploma) ☐
   5. University ☐
7. Have you ever attended road trauma first aid course? If no go to question 9
   1. Yes ☐
   2. No ☐

8. If yes where did you attend
    1. During police training
    2. On workshops
    3. Others mention…………………………
9. Have you ever cared RTI victim? If no go to question 10
    1. Yes
    2. No
10. If yes who many times?
    1. 1-2 times
    2. 3-6 times
    3. More than 6 times

PART B: Knowledge: Please answer by checking in space provided.

11. After car crash accident priority should be given to the
    a. Victim with breathing problems
    b. Victim who is bleeding heavily
    c. Victim with a fracture
    d. Confused patient.
12. What will you do to open an obstructed airway of the victim
    a. Uncover the victim by removing clothes especially around the chest.
    b. Depopulate the area so the victim can get fresh air
    c. Perform modified chin lift and head tilt
    d. Fan the patient to increase ventilation.
13. Which one is a recommended position for victim who sustains traumatic RTI at the scene?
    a. Laying on the back
    b. Laying on the stomach
    c. Laying on the side
    d. Upright position.
14. The best and effective way to manage bleeding is by:
    a. Put some salt to the wound
    b. Apply ice directly to the wound
    c. Apply direct pressure to the wound
    d. Cover the wound with gauze.

15. If the victim sustains fracture during RTI: what will be the initial care?
   a. Immobilize victim’s bone and apply splint ☐
   b. Push the bone back in place ☐
   c. Tie the bone tightly together ☐
   d. Stretch/ pull the bone to align. ☐

PART C: Reported Practice

Scenario: A car hit a motorcycle which was crossing the road. The motorcyclist (Juma) was thrown several meters away from the road into a ditch. Meanwhile the car lost balance and crashed into the light pole, the driver managed to escape. Assume you’re the only person at the scene with the victim who is unresponsive and bleeding profusely from a fractured left leg. From the given scenario**: Please write your response in space given in question 15-18**

16. How would you prevent cross infection during the process of helping the victim?
   ………………………………………………………………………………………
   ………………………………………………………………………………………
17. If The victim is not breathing how would you help him?
   ....
   ....
18. How would stop bleeding from a fractured leg of the victim?
   ………………………………………………………………………………………
   ………………………………………………………………………………………
19. How would you initially care for a fractured leg?
   ………………………………………………………………………………………
   ………………………………………………………………………………………

PART D: Attitude

Do you think the following statements are suitable to describe your views on provision of first aid to RTI victims? Please check where applicable.

20. I believe it is my responsibility to provide trauma first aid to RTI victims
   Strongly agree ☐ Agree ☐ Disagree ☐ Strongly disagree. ☐
21. I believe trauma first aid to RTI victims should be initiated immediately by first responder.
   Strongly agree ☐ Agree ☐ Disagree ☐ Strongly disagree. ☐
22. I'm willing to provide post-crash first aid
   Strongly agree ☐ Agree ☐ Disagree ☐ Strongly disagree. ☐
23. I believe providing post-crash first aid will increase survival chance
   Strongly agree ☐ Agree ☐ Disagree ☐ Strongly disagree. ☐

………………End of the questionnaire………………
